# Sexual identity inequalities in the co-occurrence of poor mental health and health risk behaviours—a national cross-sectional study

**DOI:** 10.1186/s12916-025-04236-2

**Published:** 2025-07-09

**Authors:** Amal R. Khanolkar, Alexis Karamanos, Laia Becares

**Affiliations:** 1 Department of Population Health Sciences, Guy’s Campus, King’s College London, London, UK; 2 Department of Global Health and Social Medicine, King’s College London, Strand Campus, King’s College London, London, UK

**Keywords:** Sexual minority, LGBTQ + health, Mental health, Health behaviours, Self-harm, Attempted suicide, Adolescence

## Abstract

**Background:**

Mental health problems (MHP, like depression/anxiety) and health and risk behaviours (HRBs) are more common among sexual minority adolescents (SMA) than in heterosexual peers. Limited studies have examined the co-occurrence of poor mental health and HRBs, if co-occurrence differs by sexual identity, and associated risks for self-harm and attempted suicide in adolescents.

**Methods:**

This study included 10,233 adolescents aged 17 years (51% female/11% sexual minority) from the UK-wide Millennium Cohort Study. Sexual identity, MHP, seven HRBs (like regular smoking, drug use and sexual risk behaviour), self-harm and attempted suicide were self-reported. MHP were assessed using the strengths and difficulties questionnaire [SDQ] emotional symptoms subscale for depression/anxiety. We assessed associations between sexual identity and co-occurrence of MHP and HRBs using multinomial logistic regression. We estimated predicted probabilities for self-harm or attempted suicide based on sexual identity and MHP-HRB co-occurrence status using logistic regression models with appropriate interaction terms (between sexual identity and MHP-HRB co-occurrence status variables).

**Results:**

MHP prevalence was higher in gay/lesbian (48%) and bisexual (49%) adolescents compared to heterosexual peers (19%). Self-harm (bisexual, 64%; gay/lesbian, 53%; heterosexual, 19%) and attempted suicide (bisexual, 24%; gay/lesbian, 17%; heterosexual, 6%) prevalence were higher in SMA compared to heterosexual peers. Gay/lesbian and bisexual adolescents consistently had higher probability for MHP-HRB co-occurrence compared to heterosexual peers (example, for gay/lesbian individuals: RRR 3.16 [95% CI 2.1–4.68] for MHP-1HRB, RRR 3.54 [95% CI 2.06–6.08] for MHP- ≥ 3HRB, for bisexual adolescents: RRR 2.44 [95% CI 1.85–3.20] for MHP-1HRB, RRR 4.11 [95% CI 2.99–5.66] for MHP- ≥ 3HRB). MHP-HRB co-occurrence and sexual minority identity were independently associated with greater odds for self-harm or attempted suicide. SMA had higher probabilities of reporting self-harm than heterosexual peers with the same level of MHP-HRB co-occurrence. For example, 37.2% of heterosexual adolescents with MHP-1HRB reported self-harm. Corresponding numbers were twice as high in bisexual (75.7%) and gay/lesbian (77.9%) individuals. Similarly, 58.3% of heterosexual adolescents with MHP- ≥ 3HRBs reported self-harm, increasing to 84.6% in bisexual and 83.8% in gay/lesbian peers.

**Conclusions:**

SMA are more likely to experience MHP-HRB co-occurrence, which is associated with substantially higher risks for self-harm and attempted suicide compared to heterosexual peers. Findings highlight the need for better public health policies to address MHP and associated comorbidities to reduce sexual identity-related health inequities in adolescence.

**Supplementary Information:**

The online version contains supplementary material available at 10.1186/s12916-025-04236-2.

## Background

Mental health problems like depression and anxiety among adolescents are a significant public health concern. The 2023 Mental Health of Children and Young People in England survey found that almost one in four (23.3%) adolescents aged 17 to 19 years had a probable mental health disorder (like symptoms of depression/anxiety), with rates higher among girls (31.6%) than boys (15.4%) [1]. Further, 11% of young people aged 17–24 years with a probable mental health disorder had self-harmed. In total, 6069 suicides were recorded in England and Wales in 2023 (or 11.4 deaths per 100,000), the highest rate since 1999 [2]. Suicide in young people is a global health problem, being the fourth leading cause of death among 15–19-year-olds with the suicide rate among young people aged 15–24 years being 7.7/100,000 [3]. Rates of self-harm have fallen over recent years in England (example, emergency hospital admissions for intentional self-harm reduced from 195.9 per 100,000 in 2015/16 to 117 per 100,000 in 2023/24). However, capturing rates of self-harm is complicated and reported statistics are likely a significant underestimate [4]. There are also differences in hospital-reported admissions and self-report data, with the latter showing increasing trends in self-harm especially in women and younger adults [5].

Adolescents who identify as non-heterosexual (hereon sexual minority adolescents, SMA) consistently report substantially higher levels of substance use and risk behaviours (like sexual risk behaviour [6], gambling, and drug and alcohol misuse) than their heterosexual peers [7, 8]. The most studied risk behaviours in SMA include smoking, drug and alcohol misuse, and sexual risk behaviour (example, sex without contraceptives or protection) [8, 9]. Evidence indicates both an earlier age of initiating use and using many substances concurrently in SMA compared to heterosexual peers [9, 10]. Studies also report differences by gender and SMA sub-groups [9]. For example, bisexual and female sexual minority (SM) individuals report higher smoking and substance use than SM men [7, 11]. These inequalities are significant as persistent substance use and misuse is associated with greater morbidity and a higher likelihood of addiction later in life [12]. Emerging evidence indicates that gambling (including high-risk problem gambling) is more common in SM individuals compared to heterosexual peers and is associated with lower self-esteem and poorer mental health [13]. Certain health and risk behaviours (HRBs) are more likely to cluster or co-occur and are associated with adverse health and social outcomes; example, individuals who gamble frequently are also more likely to report substance abuse [13].

Further, some HRBs impair judgement and increase the likelihood of other HRBs, example, SMA who misuse illicit drugs are more likely to engage in sexual risk behaviour, have higher numbers of sex partners, experience sexually transmitted diseases, violence, poor mental health, and homelessness [14, 15]. 

A key driver of higher risk of HRBs in SMA is previous experience of victimisation [16, 17]. Several SM-related stressors (social and psychological) like stigma and discrimination differentially impact the lived experiences, mental health, resilience and negatively influence HRBs in SMA [18–20]. This is described in the seminal ‘Minority stress theory’, the foundational framework to explain health disparities in SM groups [21]. SM individuals experience unique and long-term stress processes associated with their minority identities (example, prejudice, victimisation, internalised homophobia) because of hostile and stressful environments which subsequently increases risk for a wide range of mental health problems including self-harm and suicide. The higher levels of HRBs in SMA are largely thought to be a coping mechanism for having to live with minority-related stressors [20]. HRBs can be further exacerbated in individuals with multiple minority identities (sexual, gender, ethnic) as each identity, in isolation and as they intersect, has a different experience of stress [22]. Thus, individuals with multiple minoritised identities are differentially impacted by various adverse forms of discrimination and stigma associated with each and multiple intersecting identities, as explained in the Intersectionality Framework theory [23]. Some studies indicate higher rates of HRBs in individuals with ≥ 2 minority identities [22, 24], while others have found that ethnic and SMA were less likely to report HRBs compared to their White SMA peers [25].

In general, substance use (cigarette smoking and alcohol consumption) has been decreasing among youth of all sexual orientations in recent decades [26]. However, this decrease is not the same between SMA and heterosexual peers, and SM inequalities in HRBs persist by SMA subgroup, substance type and gender. For example, studies from the US have found that cigarette smoking has decreased in heterosexual and gay/lesbian youth but not as much in bisexual peers [27]. Other studies report that while alcohol consumption has decreased among all youth, rates have not decreased as much in SMA, who also report more hazardous drinking patterns [28].

A substantial body of evidence highlights the increased risk for poorer mental health in SMA, consistent with multiple indicators [22, 25, 29, 30]. Across adolescence and adulthood, the prevalence of common mental health problems like depression, anxiety and self-harm are 3–5 times higher in SM youth relative to their heterosexual peers [31]. Mental health inequalities result from the hostile environment in which SM people live, including increased stress, marginalisation, and denial of their identities and rights. Studies on SM health have adopted a syndemic framework to understand stark and persistent SM health inequities emerging from psychosocial hostility and socioeconomic adversity experienced by SM people across socio-political contexts, and this has been found to be particularly relevant for younger SM people [32]. In countries with some of the most progressive LGBTQ + rights and laws including the US and UK, SM individuals including adolescents and young adults continue to report high levels of abuse including bullying and physical harassment, and worryingly, the majority who experience abuse do not report it or receive no help when it is reported [33–36]. These ‘hostile environments’ for SM young people exist in schools, universities, community spaces, places of worship and public transport and can be exacerbated in rural areas and smaller communities [34, 35]. In addition to abuse and victimisation, the lack of support for SM individuals in educational institutions, including no role models, experiencing LGBTQ + hate crimes and not being accepted as SM, all contribute to worse mental health and higher suicide risk among SM individuals [35, 37]. These ‘hostile environments’ perpetuate the necessary factors required for sustained health inequities experienced by SMA and young people [37]. These facts suggest that despite substantial and positive legal and societal changes, SM individuals continue to face stigma and discrimination which impact their health. Further, in the UK, nearly half of SM individuals who answered the National LGBT survey said they did not discuss their sexuality with healthcare staff, and 50% found it difficult to access mental health services [38]. Of significant concern is that the majority of LGBTQ + specific mental health support is not provided by the mainstream state-funded healthcare services (NHS) but in fact by community organisations and charities [39].

A smaller number of studies have examined associations between mental health problems and co-occurring HRBs in SMA and young people. Most studies are conducted in the USA, and often in relation to the most commonly studied HRBs [7, 13, 20, 40]. Studies on HRBs in SMA in the UK are limited. The few studies to date have reported significantly higher levels of HRBs in SMA compared to heterosexual peers, but these studies are limited by combining all SM subgroups together, examining a limited number of HRBs or regional study samples [25, 41]. Even fewer studies have investigated HRBs in conjunction with co-occurring mental health problems. A study on adolescents aged 14 found a strong network between SM identity, depression and victimisation which were indirectly linked to substance use. In the UK, there is limited knowledge on sexual identity-related differences in the co-occurrence between common mental health problems (like depression and anxiety) and HRBs in later adolescence. Further, whether adolescents living with co-occurring common mental problems and HRBs are more likely to report acute mental health problems like self-harm and suicidality and how this varies by sexual identity remains unexplored.

We explored sexual identity inequalities in (1) reporting the number of HRBs, (2) the co-occurrence between mental health problems (depression and anxiety) and HRBs and (3) whether having co-occurrence was associated with increased probabilities for self-harm and attempted suicide in a large sample of British adolescents aged 17 years who are part of the ongoing Millennium Cohort Study.

## Methods

### Study design and participants

The Millennium Cohort Study (MCS) is a UK birth cohort study following the lives of 19,519 children born at the start of the new millennium (2000–2002) [42, 43]. Study participants have been followed over seven sweeps to date (9 months, 3, 5, 7, 11, 14 and 17 years). Detailed information on the study, sampling and survey design can be found at: https://cls.ucl.ac.uk/cls-studies/millennium-cohort-study/. Briefly, a nationally representative birth cohort from across the UK were recruited to the MCS and included children living in non-household situations and those not born in the UK but lived in the country at recruitment. The sampling strategy was to recruit 100% of eligible children living in geographically defined areas of residence (the boundaries of electoral wards as defined before the 2001 national census) during the eligible period. A stratified cluster sampling design was used to ensure adequate representation of families living in disadvantaged areas and from ethnic minority groups and the overall response rate was 72%. Specific sub-groups of the population were intentionally over-sampled, namely children living in disadvantaged areas, children of ethnic minority backgrounds and children growing up in the smaller nations of the UK (sample weights ensure national representativeness). In total, 14,496 families were invited to participate in the age 17 sweep. Of this number, 10,625 (73.3%) families and 10,345 (71.4%) adolescents were successfully interviewed. This cross-sectional study included *N* = 10,233 children (98.9% of the eligible sample) who attended the age 17 sweep (in 2018–2019). Attrition at age 17 sweep was predicted by single-parent families, lower-income occupation and lower educational level, Black ethnicity and male sex. The STrengthening the Reporting of OBservational studies in Epidemiology (STROBE) were followed in the reporting of this manuscript.

### Measures

#### Sexual identities

Based on self-reported answers on sexuality (from eight options listed in Additional File 1: Table S1), participants were categorised into the following: (1) Completely heterosexual, (2) Bisexual and (3) Gay or lesbian. Those who answered ‘other’, ‘do not know’ and ‘preferred not to say’ were excluded (*N* = 154). Individuals who identified as mainly heterosexual were also excluded from this study. While mainly heterosexual individuals have increased risk for mental health problems compared to heterosexual peers, there is ongoing debate on whether they should be considered a separate sexual identity category or combined with bisexual individuals, especially among adolescents. For this study, we included those who identified as completely heterosexual, bisexual and gay/lesbian.

#### Mental health (MH)

In this study, MH refers to symptoms of depression and anxiety. The Strengths and Difficulties Questionnaire (SDQ) is a commonly and widely used brief screening tool comprising 5 subscales (with each subscale including 5 items for a total of 25 items) that assess a range of behavioural and emotional problems in children. The SDQ is used to assess positive and negative attributes in children over the previous 6 months. In this study, we used the SDQ emotional symptoms subscale (SDQ-E) comprising 5 items or questions (for e.g. are you often unhappy?) relating to depression, worry, fear, nervousness and somatic symptoms, which assesses symptoms of psychological distress in the preceding 6 months. The SDQ-E is widely validated and *routinely used to capture symptoms of depression and anxiety* in the general population and clinical settings [44]. Further, the SDQ-E measure has been validated against diagnostic measures of depression or anxiety disorders and is an established screening tool to distinguish those individuals who meet the diagnostic criteria for depression and anxiety from those who do not [45]. For each of the 5 items, participants could respond with not true, somewhat true or certainly true (scored 0, 1 and 2, respectively). Total scores were calculated, ranging from 0 to 10, with higher scores indicating more emotional problems (depression and anxiety). Total scores were categorised into a binary variable based on recommended cut-off points indicating participants with ‘close to average’ (< 6) vs. ‘high/very high levels’ (≥ 6) of difficulties [46]. The internal consistency of the SDQ emotional symptoms subscale was assessed using Cronbach’s alpha and was found to be acceptable (*α* > 0.8).

### Outcomes of interest

Health and risk behaviours were seven in number and included current smoking, frequent alcohol consumption (in the past year), frequent cannabis use (past year), sex without contraception or protection, lack of physical activity in the previous week, antisocial behaviour in the previous year (for e.g. pushed or shoved/hit/slapped/punched someone, hit someone with or used a weapon etc.) and any form of gambling in the preceding month (for e.g., have you spent your own money in a betting shop or fruit machines?). Frequent alcohol consumption and frequent cannabis use were operationalised as > 5 times and > 4 times, respectively [25]. Each HRB was coded as a binary variable (No vs. Yes). Based on these seven binary HRB variables, we created a cumulative variable which indicates the total number of HRBs each individual reports (number of HRBs with a range of 0 to 7, with higher values indicating a greater number of HRBs reported by an individual). This continuous variable was then categorised into: 0 HRBs, 1 HRB, 2 HRBs and ≥ 3 HRBs.

### Mental health (MH) problems and HRB co-occurrence (MH-HRB) indicator

We created a categorical variable which grouped study participants into one of 4 categories indicating their MH-HRB co-occurrence status: (1) individuals without co-occurrence, only MH problems *or* only HRBs (reference category), (2) MH problems and 1 HRB, (3) MH problems and 2 HRBs and (4) MH problems and ≥ 3 HRBs. Adolescents with either only MH problems or only reporting HRBs were combined with the category of no MH-HRB co-occurrence.

Self-harm (in the previous year) and attempted suicide (anytime previously, i.e. lifetime) were reported via questionnaires. Participants were asked about self-harming actions (like cutting/stabbing, burning, bruising/pinching, taking an overdose of tablets and pulling out hair). We created binary variable indicating no self-harm vs any kind of self-harm. Attempted suicide was asked with the question: ‘’Have you ever hurt yourself on purpose to end your life?’’ Responses were categorised as no vs yes.

### Covariates

Covariates included sex assigned at birth (hereafter sex), ethnicity and parental income. Parents (or guardians) reported participant’s ethnicity at age 3 (original categories in Additional File 1: Table S1) which was available for over 95% of participants. Information on ethnicity from subsequent sweeps (ages 7, 11 and 14) was used to replace any missing ethnicity at age 3. Subjects were grouped into either (1) White (ethnic majority) and (2) EM (mixed-ethnicity, South Asian, Black and ‘other’). The ‘other’ group included participants from Asia (excluding South Asia), the Middle East and South America.

Complete details, the original questions, all component items of the MH scale and all health-related indicators including HRBs and how they were categorized or operationalised for analysis are listed in Additional File 1: Table S2.

### Statistical analysis

#### Missing data and multiple imputation

A detailed description of attrition in the cohort is provided elsewhere [47]. Missing data per variable ranged from 2.4% to 62%. For the vast majority of variables, missing data was < 5%. Variables with greater missingness included alcohol frequency (24%) and gambling (34%). The complete data sample included 6494 participants with no missing data. Missing exposure, covariate and outcome data was addressed through multiple imputation using chained equations under the assumption that data were missing at random (MAR). This was suitable given that observed characteristics like ethnicity and parental income were independent predictors of missingness (i.e. ethnic minority and socioeconomically disadvantaged participants were more likely to have missing information across ≥ 1 analysis parameters). To predict missing data, the imputation model integrated all HRBs and covariates, and auxiliary variables like parental highest educational qualifications, the four other subscales of the SDQ, indicators of mental wellbeing, BMI, general health, physical health, victimisation, doctor-diagnosed depression along with combined sample and attrition weights to strengthen the imputation model [48, 49]. Estimates were obtained by pooling results across 25 imputed data sets using the Rubin rules, and assessment of Monte Carlo errors suggested this was a suitable number of imputations [50]. The resulting imputed analytical sample included 10,223 participants.

We first estimated the proportions of participants across all variables of interest by sexual identity. Multinomial logistic regression was used to assess associations between the following: (1) sexual identity and number of reported HRBs (1, 2 or ≥ 3), (2) sexual identity and poor MH-HRB co-occurrence and (3) logistic regression was used to estimate associations between poor MH-HRB co-occurrence, sexual identity and risk of self-harm (or attempted suicide). We first ran logistic regression models without interaction terms to examine the independent effects on MH-HRB co-occurrence and sexual identity on self-harm (or attempted suicide). Further, to assess whether the probability of self-harm (or attempted suicide) differed by MH-HRB status and sexual identity, we also ran logistic regression models with self-harm (or attempted suicide) as the outcome and included interaction terms between sexual identity and MH-HRB co-occurrence variables.

The multinomial logistic regression model calculates the relative risk ratio (RRR), which is the ratio of two relative risks (derived from the exponentiated multinomial logit coefficient) and is interpreted for a unit change in the predictor variables. Further information on interpretation of RRR (which can also be interpreted as conditional odds ratios) is provided in the relevant Stata technical bulletin [51].

The predicted probabilities for self-harm (and attempted suicide) for all possible combinations of the levels of MH-HRB co-occurrence and sexual identity indicators (i.e. nine categories generated by the interaction term between the two variables) were estimated using the ‘*margins*’ command in Stata. This generates adjusted predictive margins or the probability for self-harm/attempted suicide in each category of interest. Predicted probabilities were visualised (by creating interaction plots using the ‘*marginsplot*’ command) to aid understanding of the interaction terms between the MH-HRB and sexual identity variables. All three models described above were first run without any covariates (crude models or Model 1) and then run including adjustment for sex at birth, ethnicity and parental income (Model 2).

All models above were also run stratified by sex at birth to assess whether associations differed between males and females.

All estimates were weighted using appropriate sample and attrition weights and computed using Stata 18 (StataCorp, College Station, TX). Subgroup sample sizes were estimated from imputed proportions, weighted, and rounded and so may not sum to totals.

## Results

Tables 1 and 2 present descriptive statistics of the study sample. Of the 10,223 participants, 11% identified as bisexual or gay/lesbian. MH problems (symptoms of depression and anxiety) were more than twice as high in bisexual (49.4%) and gay/lesbian (48.5%) compared to heterosexual (18.9%) adolescents. Bisexual (19.4%) and gay/lesbian (17.1%) adolescents were more likely to report ≥ 3 HRBs compared to heterosexual (12.9%) peers. Overall, 3.4% of participants reported having poor MH and ≥ 3 HRBs. There were significant differences in proportions of participants reporting co-occurring poor MH and 2 HRBs and ≥ 3 HRBs (example, 10.3% of bisexual adolescents reported poor MH and ≥ 3 HRBs compared to 2.6% of heterosexual adolescents). Similarly, proportions of individuals reporting self-harm or attempted suicide were much higher in those with co-occurring poor MH and ≥ 3 HRBs.

**Table 1:** Prevalence of poor mental health, health and risk behaviours (HRB) by sexual identity in 10,233 adolescents aged 17 years from the Millennium Cohort Study

Variables of interest	Heterosexual		Bisexual		Gay/lesbian		Full sample	
	%	95% CI	%	95% CI	%	95% CI	%	95% CI
**Sexual identity**	88.9	88.3-89.6	8.1	7.4–8.6	2.9	2.6–3.4		
**Sex at birth**								
Male	51.4	50.3–52.4	24	20.8–27.2	44.9	38.9–50.9	48.9	47.9–49.9
Female	48.6	47.6–49.7	76	72.8–79.2	55.1	49.1–61.1	51	50–52
**Ethnicity**								
White	79.7	78.9–80.5	90.3	88.1–92.4	91.5	88.1–94.9	80.9	80.1–81.7
Non-White	20.3	19.5–21.2	9.7	7.5–11.9	8.5	5.1–11.9	19.1	18.3–19.8
**Poor mental health (yes)**	18.9	18.1–19.8	49.4	45.7–53.1	48.5	42.1–54.9	22.3	21.5–23.1
**Health risk behaviours**								
* 1 HRB*	32.7	31.7–33.4	31.9	28.6–35.4	37.3	31.2–43.4	32.8	31.9–33.7
* 2 HRB*	15.9	15.2–16.7	18.3	15.5–21.1	18.1	13.3–22.8	16.2	15.5–16.9
* ≥3 HRB*	12.9	12.2–13.6	19.4	16.5–22.3	17.1	12.5–21.6	13.5	12.8–14.2
**Self-harm**	18.9	18.1–19.8	63.7	60.1–67.3	53.1	46.9–59.2	23.6	22.7–24.4
**Attempted suicide**	5.8	5.3–6.3	24	20.7–27.3	17.5	12.6–22.3	7.6	7.1–8.2

Mental health (assessed using the Strengths and Difficulties Questionnaire [SDQ]; emotional symptoms subscale)

**Table 2:** Prevalence of co-occurring poor mental health and health and risk behaviours (HRB) by sexual identity in 10,233 adolescents aged 17 years from the Millennium Cohort Study

MH-HRB co-occurrence	Full sample %	95% CI		Sexual identity	%	95% CI	
No co-occurrence	85.6	84.9	86.3	Heterosexual	88	87.3	88.7
				Bisexual	65.9	62.4	69.3
				Gay/lesbian	67.2	61.4	73.1
MH and 1 HRB	7.2	6.7	7.7	Heterosexual	6.1	5.6	6.6
				Bisexual	14.8	12.3	17.4
				Gay/lesbian	17.3	12.7	2.2
MH and 2 HRB	3.9	3.5	4.2	Heterosexual	3.3	2.9	3.6
				Bisexual	9	6.9	11.1
				Gay/lesbian	7.8	4.4	11.2
MH and ≥3 HRB	3.4	3.0	3.7	Heterosexual	2.6	2.3	2.9
				Bisexual	10.3	8.1	12.4
				Gay/lesbian	7.7	4.4	10.9

*MH: *Mental health (assessed using the Strengths and Difficulties Questionnaire [SDQ]; emotional symptoms subscale), *HRB: *Health and risk behaviours (antisocial behaviour, current smoker, frequent alcohol consumption, frequent cannabis use, sex without contraception or protection, no exercise, gambling)

### Associations between sexual identity and number of reported HRBs (Table 3)

In general, SM adolescents (bisexual and gay/lesbian individuals) were significantly more likely to report 1, 2 or ≥ 3 HRBs compared to their heterosexual peers. We did not find any differences in reporting of HRBs between bisexual and gay/lesbian individuals (i.e. 95% CIs overlapped for most estimates). Adjustment for covariates (ethnicity, parental income and sex) did not significantly change estimates. Both bisexual (adjusted RRR 2.06, 95% CI 1.58–2.70) and gay/lesbian individuals (RRR 1.59, 1.02–2.48) were significantly more likely to report ≥ 3 HRBs compared to their heterosexual peers.

**Table 3:** Associations between sexual identity and health and risk behaviours (HRB) in 10,223 adolescents from the Millennium Cohort Study. Estimates are from multinomial logistic regression models

	Model 1		Model 2	
	RRR	95% CI	RRR	95% CI
***No HRB***	Ref		Ref	
***1 HRB***				
Heterosexual	Ref		Ref	
Bisexual	1.21	0.96, 1.53	**1.26**	**1.00, 1.59**
Gay/Lesbian	**1.55**	**1.06, 2.26**	**1.57**	**1.07, 2.30**
**Sex at birth**				
Male			Ref	
Female			**0.87**	**0.78, 0.98**
**Ethnicity**				
White			Ref	
Ethnic-minority			1.0	0.85, 1.17
**Parental income**				
Quintile 1			1.14	0.97, 1.35
Quintile 2			1.09	0.92, 1.30
Quintile 3			0.93	0.79, 1.11
Quintile 4			0.97	0.82, 1.13
Quintile 5 (highest income)			Ref	
***2 HRBs***				
Heterosexual	Ref		Ref	
Bisexual	1.28	0.98, 1.68	**1.35**	**1.03, 1.78**
Gay/Lesbian	1.42	0.91, 2.21	1.42	0.91, 2.23
**Sex at birth**				
Male			Ref	
Female			**0.73**	**0.63, 0.85**
**Ethnicity**				
White			Ref	
Ethnic-minority			**0.6**	**0.48, 0.76**
**Parental income**				
Quintile 1			1.01	0.82, 1.25
Quintile 2			1.01	0.82, 1.25
Quintile 3			0.84	0.68, 1.05
Quintile 4			0.8	0.64, 0.99
Quintile 5 (highest income)			Ref	
***≥3 HRBs***				
Heterosexual	Ref		Ref	
Bisexual	**1.77**	**1.36, 2.30**	**2.06**	**1.58, 2.70**
Gay/Lesbian	**1.57**	**1.02, 2.43**	**1.59**	**1.02, 2.48**
Sex at birth				
Male			Ref	
Female			**0.51**	**0.44, 0.60**
**Ethnicity**				
White			Ref	
Ethnic-minority			**0.38**	**0.28, 0.50**
**Parental income**				
Quintile 1			**1.30**	**1.03, 1.65**
Quintile 2			**1.30**	**1.03, 1.65**
Quintile 3			1	0.80, 1.26
Quintile 4			1.04	0.84, 1.30
Quintile 5 (highest income)			Ref	

Model 2: adjusted for sex at birth, ethnicity and parental income. Text in bold indicates estimates for which 95% CI do not contain 1

### Associations between sexual identity and co-occurring poor MH and HRBs (Fig. 1 and Additional File 1: Table S3)

**Fig. 1:**
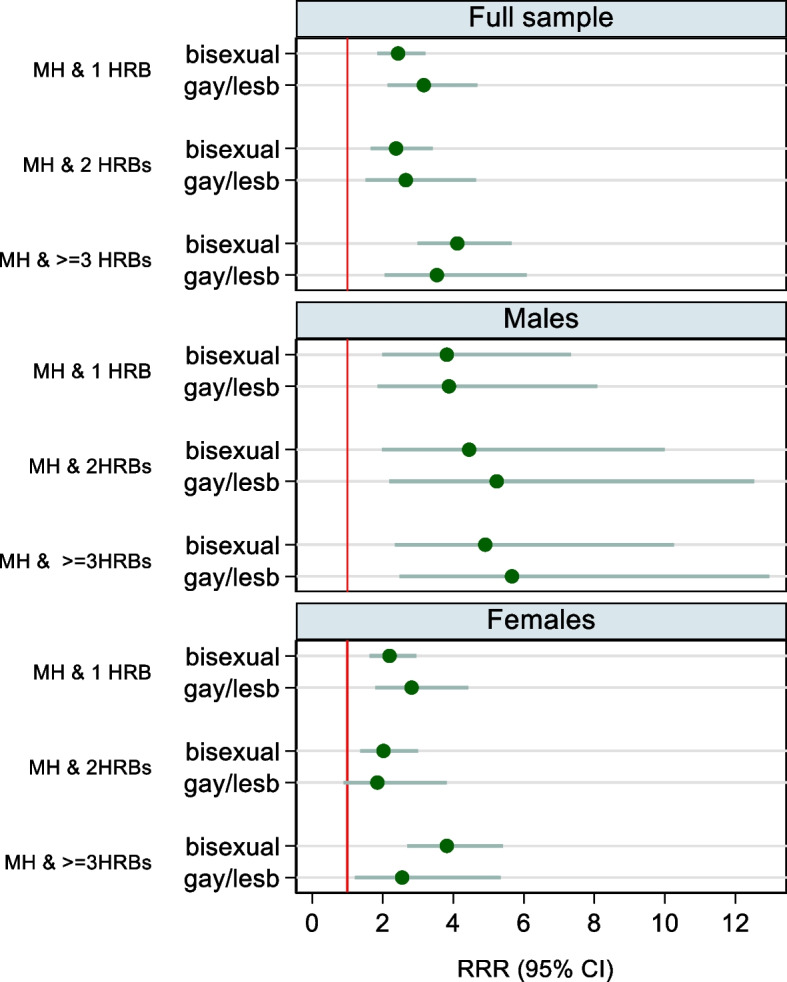
Associations between sexual identity and co-occurring poor mental health and health and risk behaviours (HRBs) in 10,223 adolescents from the Millennium Cohort Study. Estimates are from multinomial logistic regression models (presented in Supplemental Tables 3 and 8). Reference categories: Heterosexual individuals and those with no MH and HRB co-occurrence

We found consistent and significant associations between sexual minority identity and co-occurring poor MH and HRBs. Compared to heterosexual individuals, bisexual and gay/lesbian individuals were consistently more likely to be in all three categories of poor MH and co-occurring HRBs (example, adjusted RRR 4.11 [2.99–5.66] and adjusted RRR 3.54 [2.06–6.08] for MH and ≥ 3 HRBs for bisexual and gay/lesbian individuals respectively). Adjustment for covariates (ethnicity, parental income and sex) only marginally attenuated the estimates.

### Associations with self-harm and attempted suicide (Additional Files 1: Tables S4 and S5)

MH-HRB co-occurrence and sexual identity were independently associated (and after mutual adjustment) with increased odds for self-harm or attempted suicide. In logistic regression models *without* interaction terms, MH-HRB co-occurrence was significantly associated with increased odds for self-harm and attempted suicide. Further, there was a gradient in odds for self-harm (or attempted suicide) with increasing number of poor MH and co-occurring HRBs. Example, the odds for self-harm increased progressively from adjusted OR 3.14 (2.60–3.79) to OR 3.63 (2.80–4.72) and OR 7.01 (5.14–9.56) for 1 HRB, 2 HRBs and ≥ 3HRBs, respectively, with co-occurring poor MH. Bisexual (adjusted OR 6.0, 4.81–7.49) and gay/lesbian (adjusted OR 3.88, 2.79–5.38) had significantly increased odds for self-harm compared to heterosexual peers. A similar pattern was observed between co-occurring MH-HRB and sexual identity and attempted suicide.

The ORs for self-harm or attempted suicide for the different categories of MH-HRB co-occurrence (i.e. main effects) in logistic regression models *with* interaction terms (between sexual identity and MH-HRB variables) are for the reference category of heterosexual adolescents. This group of adolescents had significantly increased odds for self-harm or attempted suicide with a gradient (ORs increased with increasing number of HRBs and co-occurring poor MH). The ORs for self-harm or attempted suicide for bisexual and gay/lesbian adolescents (main effects) are for those without MH and HRB co-occurrence (reference category). These adolescents had significantly increased odds for both outcomes; for example, bisexual (adjusted OR 6.64 [5.17–8.51]) and gay/lesbian (adjusted OR 3.83 [2.61–5.62] adolescents had substantially higher odds for self-harm compared to heterosexual peers, among adolescents without co-occurrence. The estimates for interaction terms between MH-HRB and sexual identity variables are explained using predicted probabilities below.

### Predicted probabilities for self-harm and attempted suicide based on sexual identities and MH-HRB co-occurrence status (Additional File 1: Table S6 and Fig. 2)

**Fig. 2:**
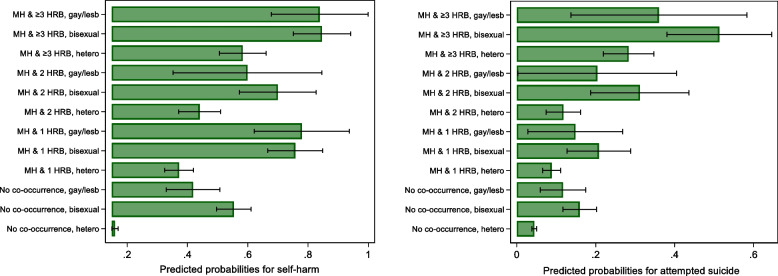
Predicted probabilities for self-harm (and attempted suicide) based on poor mental health and health and risk behaviours (HRBs) co-occurrence and sexual identities in 10,223 adolescents aged 17 years from the Millennium Cohort Study. Estimates are based on multivariable logistic regression models (adjusted for sex, ethnicity and parental income, Table 4)

Predicted probabilities can be interpreted as the proportions of the sample presenting with the outcome of interest after accounting for confounders. Irrespective of sexual identity, adolescents with co-occurring poor MH and HRBs had increased probabilities of self-harm or attempted suicide (with probabilities increasing progressively with increasing number of HRBs). For example, 41.7% of adolescents with MH and 1 HRB reported self-harm increasing to 61.3% for those with MH and ≥ 3 HRBs. Greater proportions of SM adolescents reported self-harm and attempted suicide compared to heterosexual peers.

However, the probabilities for self-harm and attempted suicide were even higher (or varied in strength) in SMA compared to heterosexual peers *reporting the same level of co-occurring poor MH and HRBs*. Or where individuals reported poor MH and co-occurring HRBs, the probability of self-harm and attempted suicide among SMA was higher compared to heterosexual peers. For example, 37.2% of heterosexual adolescents with MH and 1 HRB reported self-harm. Corresponding numbers were more than twice as high in bisexual (75.7%) and gay/lesbian (77.9%) individuals. Similarly, 58.3% of heterosexual adolescents with MH and ≥ 3 HRBs reported self-harm, which increased to 84.6% in bisexual and 83.8% in gay/lesbian peers.

We observed a similar pattern for attempted suicide; but findings were larger and more evident for those with MH and ≥ 3 HRBs and bisexual individuals. 30.6% of adolescents with MH and ≥ 3 HRBs reported attempted suicide compared to 10.1% for those with MH and 1 HRB. Bisexual individuals with co-occurring MH-HRBs had the greatest proportions reporting attempted suicide (20.1% for MH and 1 HRB, 31.2% for MH and 2 HRBs and 51.3% for MH and ≥ 3HRBs). On average, greater proportions of gay/lesbian individuals reported attempted suicide compared to heterosexual peers, but these differences were smaller with overlapping 95% CIs.

### Sex stratified results (Additional File 1: Tables S7, S8, S9 and S10, and Figs. 3 and 4)

**Fig. 3:**
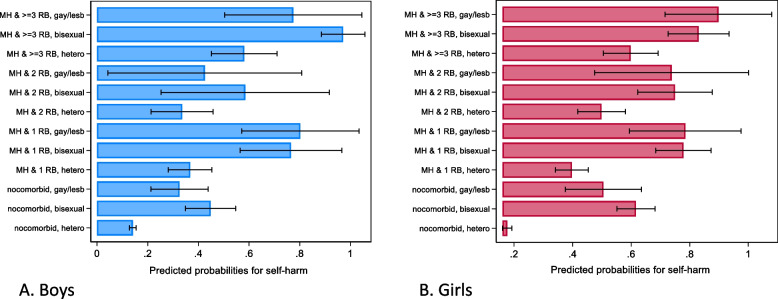
Predicted probabilities for self-harm based on poor mental health and health and risk behaviours (HRBs) co-occurrence and sexual identities in 10,223 adolescents aged 17 years from the Millennium Cohort Study. Estimates are based on multivariable logistic regression models (adjusted for ethnicity and parental income) stratified by sex

**Fig. 4:**
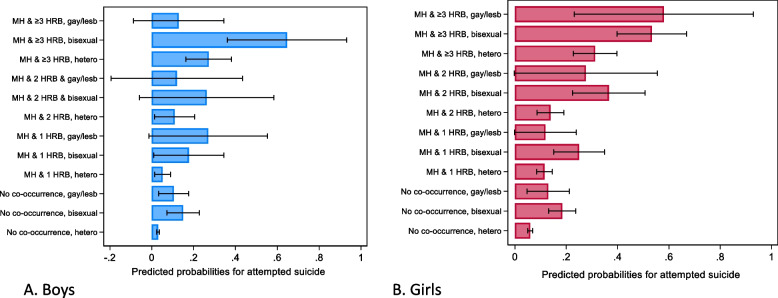
Predicted probabilities for attempted suicide based on poor mental health and health and risk behaviours (HRBs) co-occurrence and sexual identities in 10,223 adolescents aged 17 years from the Millennium Cohort Study. Estimates are based on multivariable logistic regression models (adjusted for ethnicity and parental income) stratified by sex

SMA girls were significantly more likely to report any number of HRBs compared to heterosexual girls. Findings were more consistent for bisexual girls (RRR 1.32 [1.00–1.73] for 1 HRB, RRR 1.39 [1.01–1.90] for 2 HRBs and RRR 2.32 [1.70–3.17] for ≥ 3 HRBs than gay/lesbian peers. While SMA boys had higher RRR for reporting HRBs, these were largely non-significant.

SMA boys and girls were significantly more likely to have MH-HRBs co-occurrence compared to heterosexual peers. However, on average, boys had a higher likelihood for co-occurring MH-HRBs compared to girls. This was observed for all categories of co-occurring MH-HRBs (example, for MH and ≥ 3 HRBs, boys: bisexual RRR 4.91 [2.35–10.26] and gay/lesbian RRR 5.66 [2.48–12.96] compared to girls: bisexual RRR 3.82 [2.69–5.41] and gay/lesbian RRR 2.55 [1.22–5.34]). But for all estimates, 95% CIs were largely overlapping.

Probability for self-harm and attempted suicide increased with increasing number of co-occurring HRBs and poor MH in both heterosexual boys and girls. For example, the likelihood for self-harm (heterosexual boys, adjusted OR 8.49 [4.88–14.77]; girls, adjusted OR 7.03 [4.68–10.56]) in adolescents with MH and ≥ 3 HRBs was more than twice has high compared to those with MH and 1 HRB. Both bisexual and gay/lesbian adolescents with no MH-HRB co-occurrence had statistically significant increased ORs for both self-harm and attempted suicide. The estimates for interaction terms between MH-HRB and sexual identity variables are explained using predicted probabilities below.

Heterosexual individuals without co-occurring poor MH and HRBs had the lowest probabilities for self-harm and attempted suicide in both sexes (14.1% and 17.6% for self-harm in boys and girls, respectively). Bisexual (65%) and lesbian (56.6%) girls had higher proportions reporting self-harm compared to bisexual (47.4%) and gay (35.5%) boys. For all levels of co-occurrence, greater proportions of SMA boys and girls reported self-harm than heterosexual peers. For MH and 2HRBs, greater proportions of SMA girls reported self-harm compared to SMA boys, but CIs were overlapping. Proportions of SMA with MH and ≥ 3 HRBs reporting self-harm were similar in boys and girls.

A similar pattern was observed for attempted suicide. Greater proportions of SMA boys and girls reported attempted suicide compared to heterosexual peers with the same level of co-occurrence. However, there was no observable differences between boys and girls.

## Discussion

The present study aimed to investigate sexual identity inequalities in HRBs, co-occurrence between poor MH and HRBs and whether this co-occurrence was associated with an increased probability for self-harm and attempted suicide. We found that SMA were significantly more likely to report a greater number of HRBs compared to heterosexual peers. SMA were also more likely to report co-occurring poor MH-HRBs. Further, sexual minority status and having co-occurring poor MH-HRB were independently associated with an increased likelihood of self-reported self-harm and attempted suicide (with a gradient—i.e. risk for both outcomes increased with increasing number of HRBs and co-occurring poor MH). A pertinent finding is that greater proportions of SMA reported self-harm or attempted suicide in relation to co-occurring MH-HRBs compared to heterosexual peers with the same level of co-occurrence. Differences between gay/lesbian and bisexual individuals were limited. We found no difference in proportions of gay/lesbian or bisexual individuals reporting self-harm in relation in MH-HRB co-occurrence. However, there was some evidence for greater proportions of bisexual adolescents who reported attempted suicide compared to gay/lesbian adolescents with the same level of co-occurring MH-HRB. Differences by sex were evident for some of the associations studied. Overall, male adolescents were less likely to report HRBs compared to females, regardless of sexual identity. While female SMA were more likely to report co-occurring MH-HRBs compared to heterosexual peers, their likelihood for co-occurrence was smaller than male SMA peers.

Strengths include a large sample size drawn from a nationally representative, highly studied contemporary birth cohort with rich social, health and economic data, which facilitates generalisability to the UK adolescent population. Our indicator of MH (SDQ emotional symptoms sub-scale) is widely used in studies on adolescent mental health increasing comparability with other studies. In general, missing data on all confounders and MH were relatively low (< 5%) but we did have higher levels of missing data on two HRBs, and 9% of the sample were missing data on sexual identity. We addressed missing data using the robust multiple imputation technique. Multiple imputation relies on the MAR assumption, which is not empirically verifiable [52]. Nonetheless, we increased the plausibility of the MAR assumption by including a rich set of auxiliary variables which help increase the precision of predicting missing data and minimising non-random variation in values. Additionally, the wide range of associated variables like socioeconomic, health and HRBs data and longitudinal data on BMI are powerful predictors of missing data which significantly strengthen the imputation model. We included HRBs like gambling and antisocial behaviour which are seldom studied in relation to sexual identity and MH-HRB co-occurrence.

Like most cohort studies, the MCS has significant attrition over follow-up (of the original 19,517 children, 10,757 attended the age 17 sweep). However, some studies have indicated that the MCS remains representative of its original birth cohort, and we used survey weights to adjust for attrition and ensure population-representativeness. We could not examine important factors like access to MH services, peer-group support, SM-specific issues like rejection sensitivity, internalised stigma, gender-role strain and perceived burdensomeness which are strongly linked to MH and wellbeing, and self-harm and suicidality, and are known to differ between heterosexual and SM individuals [53]. As this is a cross-sectional study, we could not disentangle the direction of association between MH-HRB and self-harm/attempted suicide. However, we hypothesise that it is more likely that low mood/depression and persistent HRBs subsequently leads to self-harm and/or attempted suicide as reported by other studies [54]. Further, the cross-sectional design precludes causality. Nonetheless, we hypothesised that for most individuals’ sexual identities or orientation are likely formulated earlier in adolescence before age 17. Including individuals with only mental health problems or only reporting HRBs in the reference category of individuals without co-occurrence might attenuate or reduce the effect estimates but this is minimal.

There are some limitations with using self-reported data. Participants may not accurately recall the frequency of certain behaviours like alcohol consumption and drug use during the past year leading to measurement error. Some participants may not want to think about and/or report distressing past events like self-harm or attempted suicide leading to an underestimation of these outcomes. Data on self-harm, attempted suicide and some HRBs were ascertained using single questions which may limit capturing nuanced behaviours. Nonetheless, these data are routinely captured using single questions in both research studies and national surveys and have shown to be robust indicators in adolescents and adults [5, 55]. Further, we do not expect that limitations of self-report of these past actions will differ between heterosexuals and SMA and consequently will not impact measuring health inequalities.

We excluded individuals who identified as ‘mainly heterosexual’ due to discussions on whether they should be in a separate sexual identity category or combined with bisexual individuals. A recent study using MCS data combined mainly heterosexual and bisexual individuals in the same group, an approach we do not recommend [56]. The term ‘mainly heterosexual’ is not commonly used in health questionnaires in the UK, and there is a strong likelihood for it to be confused with ‘bisexual’. Further, individuals unsure of their sexual identity (more likely in adolescence) may be unsure of which category to choose. Thus, for this study, we included individuals identifying as completely heterosexual, bisexual or gay/lesbian who are more likely to be sure about their sexual identity. Nonetheless, adolescents identifying as mainly heterosexual have increased risk for poor health compared to completely heterosexual peers, and we need to further examine health in this group while also understanding their similarities and differences with bisexual individuals [25]. Despite 11% of the sample identifying as SM, we had relatively small numbers of individuals with co-occurring MH-HRBs in SM subgroups, which could explain the wide confidence intervals observed for some estimates and the lack of differences between bisexual and gay/lesbian individuals. Smaller numbers also precluded us from testing for potential interactions with ethnicity and socioeconomic indicators. We were unable to examine associations in individuals who identify with other sexual minority identities like asexual, pansexual and demisexual as these were not explicit options in MCS questionnaires at age 17. Another potential limitation is the combining of gay and lesbian individuals in the same category, but we ran models stratified by sex which addresses this limitation to some extent. Future studies should also include where possible a wider range of HRBs like binge eating, excessive use of mobile phones/social media and pornography which are higher in prevalence in adolescence and SM groups.

Several studies have reported higher prevalence of HRBs (in particular substance use like regular smoking, alcohol misuse/binge drinking, drug use and sexual risk behaviour) in SM groups compared to heterosexual peers including adolescents and young adults [7, 40, 57, 58]. However, most of this research originates from the USA [58, 59]. While some studies show differences in prevalence between SM sub-groups (example, higher prevalence in bisexual and female SMA), others show conflicting results of no differences [7]. Associations with substance use have been found with different indicators of sexual identity (self-identified, sexual behaviour, and sexual attraction), but associations are stronger and more consistent with self-identified sexual identity which is used in our study [7, 58]. A systematic review focused on evidence from the UK found clustering between sexual risk behaviour, smoking, drug use and alcohol misuse (same HRBs as included in our study) was more common among young adults [60]. Most studies that have examined sexual identity related differences in HRBs in adolescents have not examined co-occurrence with poor mental health limiting comparisons. A recent study on approximately 8000 university students in Ireland found higher level of risky mental and sexual health behaviours in SM individuals compared to heterosexual peers but not for risky physical behaviours like alcohol and drug use, which contrasts with our findings [59]. Further, the authors found evidence for an ‘overlap’ between different forms of adverse health behaviours and mental health outcomes like depression and anxiety, but the methods used to evaluate co-occurring behaviours and adverse health was different from our study. Further, many studies focus on MH co-occurring with health-related behaviours (like substance use, sexual risk behaviour) or are restricted to samples comprising only SM populations, or those with a particular health condition like HIV or cancer.

Our findings of sex differences in reporting multiple HRBs (regardless of sexual identity) is in line with existing literature. We found evidence that SMA females (especially bisexual) were more likely to report any number of HRBs compared to both heterosexual peers and male SMA, which is line with previous studies [40, 61]. We advise caution in interpreting sex related differences due to smaller numbers in sex-stratified models.

The Syndemic Health Model predicates that health behaviours and conditions often co-occur and interact across the lifecourse, increasing risk for adverse health in subpopulations further compounded by environmental conditions like socioeconomic disadvantage, which perpetuate this concentration of conditions [32, 62]. This can be applied in the context of sexual minority inequalities where SMA experience multiple stressors (like stigma, discrimination, victimisation, living in a heteronormative society which contribute to a hostile environment) adding to existing internal stressors like guilt, low self-esteem and internalised stigma associated with their sexual and other identities which impact not only their mental but also physical health. This is embodied in Meyer’s minority stress theory which is extended to explain the higher levels of HRBs especially substance use in sexual minority individuals. SMA might use HRBs as coping mechanisms to deal with living with multiple stressors in an already volatile life period like adolescence. In our previous work also using the MCS data, SMA were more than twice as likely to report bullying compared to heterosexual peers, which was associated with substantially worse mental health including psychological distress, self-harm, suicidality and victimisation [33]. This highlights the continued exposure to various forms of stigma and discrimination among SMA even today despite the positive societal and legal advances for SM groups in the UK and elsewhere. Substantial evidence supports greater levels of interactions between mental, physical and sexual health and behaviours in SM individuals compared to heterosexual peers [63]. A large study on various forms of substance use by sexual identity using data on 126,463 participants from the US National Survey on Drug Use and Health found substantially higher odds for most substance use in SM adults compared to heterosexual peers [58]. But a key finding was the prevalence of substance use was even higher among SM individuals with unmet need for mental healthcare services. While we are unable to examine access to mental healthcare services in this study, previous studies have shown that long waiting times, costs and accessing the appropriate kind of mental healthcare services among other structural and systemic barriers are challenging for adolescents and young people (and their parents in the UK) [64]. Further, SMA face unique challenges as described above which require more tailored, specialist and appropriate healthcare services which are largely not currently available. In the UK, the Children and Young People’s Improving Access to Psychological Therapies (CYP-IAPT) is a programme aimed at improving access to mental healthcare, but it does not include any clear evidence-based principles and action points specifically designed for SMA.

Our findings clearly demonstrate that SMA are more likely to report a greater number of HRBs and co-occurring poor MH and HRBs which are associated with higher levels of self-harm and attempted suicide which are serious causes for concern. While we are unable to examine directionality between MH and HRBs, it is evident that a significantly higher number of SMA with poor MH are reporting higher numbers of HRBs. Our findings and the large evidence base for SM-related inequalities highlight that current policies are falling short. Substantially more needs to be done to address health inequity among SMA. Policies that more effectively address SM stressors, in combination with health promotion, awareness and equality campaigns, are needed. But these need to be addressed in multiple venues like schools, local communities and neighbourhoods, public transport and elsewhere. Age 17 is an important time for most adolescents as this is when many key life transitions like finishing secondary school, starting higher education, moving away from home, or potentially entering the labour market occur. This is also the time when adolescents transit from Child and Adolescent Mental Health Services (CAMHS) to adult mental health services, and many young individuals fall through the gaps during transition, potentially further worsening outcomes at the precise time when support is most required [65]. Thus, while there needs to be a universal approach to improving access to mental healthcare services for all adolescents, a multifaceted systems-based approach is required to address mental health and health behaviour disparities experienced by SMA. This can include sensitising school staff about these inequalities experienced by SMA, designing interventions to reduce stigmatisation and victimisation including the high levels of bullying, healthcare provider training including general practitioners who might be the first point of contact for SMA experiencing mental health problems, and broader social acceptance of sexual diversity. A key point will be to design counselling programmes that SMA find approachable and importantly LGBTQ + safe spaces, so that they may discuss HRBs and associated mental health problems without feeling stigmatised. However, these programmes need to be easily accessible like in schools or neighbourhood community centres, confidential to protect identities, and staffed by individuals aware of the unique problems faced by sexual minority groups. Funding for LGBTQ + organisations like charities which organise outreach and community programmes and are often a first point of contact for SMA should be increased.

## Conclusions

To conclude, SMA are more likely to report a greater number of HRBs and co-occurring poor MH and HRBs compared to heterosexual peers. An increasing number of HRBs with co-occurring poor MH is associated with a higher risk for self-harm and attempted suicide (with a clear gradient) in adolescents, irrespective of sexual identity. However, significantly higher proportions of SMA were more likely to self-harm or attempt suicide compared to heterosexual peers with the same level of co-occurrence. This is a stark example of sexual minority identity health inequalities in a contemporary cohort of adolescents in the UK.

## Supplementary Information

Additional file 1: Table S1. The original ethnic and sexual identity variables. Table S2. Full description of mental health and HRB used in this study. Table S3. Associations between sexual identity and co-occurrence of poor mental health and health risk behaviours. Table S4. Associations between co-occurring poor mental health and HRBs and self-harm (or attempted suicide). Table S5. Associations between co-occurring poor mental health and HRBs and self-harm (or attempted suicide), with interaction[LE1] terms. Table S6. Predicted probabilities for self-harm or attempted suicide based on poor mental health and HRBs co-occurrence and sexual identities. Table S7. Associations between sexual identity and HRBs, sex stratified models. Table S8. Associations between sexual identity and co-occurrence of poor mental health and HRBs, sex stratified models. Table S9. Associations between poor mental health and co-occurring HRBs and self-harm or attempted suicide, sex stratified models. Table S10. Predicted probabilities for self-harm or attempted suicide based on poor mental health and HRBs co-occurrence and sexual identities, sex stratified models corr90_d41ca905-05ab-4ca9-af9f-da5cb2d4798e.

## Data Availability

Data for this study is from the age 17 sweep of the Millennium Cohort Study. Data can be accessed from the UK Data Service website: https://ukdataservice.ac.uk/ (after registration and agreeing to licence terms and conditions). Syntax/code used for the analysis in this study is available on request from the corresponding author.

## Abbreviations

SMA: Sexual minority adolescents
SM: Sexual minority
HRBs: Health and risk behaviours
MCS: Millennium Cohort Study
EM: Ethnic minority
MH-HRB: Poor mental health and health and risk behaviours co-occurrence
RRR: Relative risk ratio
OR: Odds ratio
