# Supplementary material for: Sexual identity inequalities in the co-occurrence of poor mental health and health risk behaviours—a national cross-sectional study
Source: BMC Med. 2025 Jul 9;23:417. doi: 10.1186/s12916-025-04236-2 (PMC12239281; doi:10.1186/s12916-025-04236-2)
Supplement: Supplementary file 1 — Additional file 1: Table S1. The original ethnic and sexual identity variables. Table S2. Full description of mental health and HRB used in this study. Table S3. Associations between sexual identity and co-occurrence of poor mental health and health risk behaviours. Table S4. Associations between co-occurring poor mental health and HRBs and self-harm (or attempted suicide). Table S5. Associations between co-occurring poor mental health and HRBs and self-harm (or attempted suicide), with interaction[LE1] terms. Table S6. Predicted probabilities for self-harm or attempted suicide based on poor mental health and HRBs co-occurrence and sexual identities. Table S7. Associations between sexual identity and HRBs, sex stratified models. Table S8. Associations between sexual identity and co-occurrence of poor mental health and HRBs, sex stratified models. Table S9. Associations between poor mental health and co-occurring HRBs and self-harm or attempted suicide, sex stratified models. Table S10. Predicted probabilities for self-harm or attempted suicide based on poor mental health and HRBs co-occurrence and sexual identities, sex stratified models corr90_d41ca905-05ab-4ca9-af9f-da5cb2d4798e. [file 12916_2025_4236_MOESM1_ESM.pdf]

Sexual identity inequalities in the co-occurrence of poor mental health and health risk behaviours – A national cross-sectional study

Amal R. Khanolkar[1], Alexis Karamanos[1] and Laia Becares[2]

1. Department of Population Health Sciences, Guy's Campus, King's College London, London SE1 1UL, UK, 2. Department of Global Health and Social Medicine, King's College London, Strand Campus, King's College London, London, UK

Additional File 1: Supplemental data

Table S1. The original categories for ethnic- and sexual identity variables that were combined for analysis

Original categories	N (%)	Collapsed for analysis	Final N (%)
**Ethnicity**			
White	7,920 (81)	**White**	**7,920 (81)**
Mixed	290 (3)		
Indian	287 (2.9)		
Pakistani	537 (5.5)		
Bangladeshi	243 (2.4)	**Ethnic minority**	**1,869 (19)**
Black Caribbean	108 (1.1)		
Black African	219 (2.2)		
'Other' ethnic group	185 (1.9)		
**Sexual identity**			**Used in main analysis**
Completely heterosexual/ straight	7,809 (78.5)	**Heterosexual**	**7,809 (89)**
Mainly heterosexual/ straight	1,084 (10.9)		
Bisexual	649 (6.5)		
Mainly gay or lesbian	90 (0.9)	**Sexual minority**	**896 (11)**
Completely gay or lesbian	157 (1.6)		
Other (not included)	154 (1.6)		

Table S2. A detailed description of mental health, and health-related behaviours assessed at the age 17 sweep of the Millennium Cohort Study

Indicator	Question(s) in cohort member computer-assisted personal interview (CAPI), self-completion interview (CASI) or online questionnaire (CAWI)	Binary or continuous	Comments
**Mental health**			
Self-reported SDQ -emotional subscale<br><br>(Symptoms of depression & anxiety)	Complains of headaches/stomach aches/sickness<br>Often seems worried<br>Often unhappy<br>Nervous or clingy in new situations<br>Many fears, easily scared.	Continuous scale (scores ranging from 0 to 10) which was dichotomised for analysis	1. Not true<br>2. Somewhat true<br>3. Certainly true.
Self-harm	During the last year, have you hurt yourself on purpose in any of the following ways?<br>Cut or stabbed yourself<br>Burned yourself<br>Bruised or pinched yourself<br>Taken an overdose of tablets<br>Pulled out your hair<br>Hurt yourself some other way	No vs yes (any kind of self-harm)	
Attempted Suicide	Have you ever hurt yourself on purpose in an attempt to end your life?	No vs yes	
**Health risk behaviours (HRB)**			
Antisocial behaviour	Any antisocial behaviour in previous 12 months:<br>Pushed or shoved/hit/slapped/punched someone?<br>Hit someone with or used a weapon?<br>Stolen something from someone. e.g. a mobile phone, money etc.?<br>Harassed or bothered someone via mobile phone or email?<br>Sent pictures or spread rumours about someone via phone, email, social media or online?<br>Made an unwelcome sexual approach or assaulted someone sexually?	No vs yes	
Regular smoker	*Please read the following statements carefully and decide which ONE best describes you:*<br>I have never smoked cigarettes<br>I have only ever tried smoking cigarettes<br>I used to smoke sometimes but I never smoke now<br>I sometimes smoke cigarettes now but I don't smoke as many as one a week<br>I usually smoke between one and six cig cigarettes<br>I usually smoke more than six cigarettes<br>Do not know / I do not wish to answer / No answer	Never/former versus current	
Frequency of alcohol consumption	How many times have you had an alcoholic drink in the last 12 months?	<5 times vs >5times	
Binge drinking	Have you ever had five or more alcoholic drinks at a time? A drink is half a pint of lager, beer or cider, one alcopop, a small glass of wine, or a measure of spirits.	No vs yes	
Frequency of cannabis use	In the past year how many times have you taken cannabis?	<4 times vs >4 times	
Sex without protection/ contraception	Do you or any partner regularly use any of these forms of contraception or protection when having sex together?	No vs yes	
Exercise in previous week	On how many days in the last week did you do a total of at least an hour of moderate to vigorous physical activity?<br>By moderate to vigorous we mean any physical activity that makes you get warmer, breathe harder and makes your heart beat faster, e.g. riding a bike, running, playing football, swimming, dancing, etc.	None vs. any amount	

Gambling	Have you spent any of your own money on any of the following in the past 4 weeks? We want to know about games you played yourself.<br>Fruit machines?<br>Placing a private bet for money (e.g. with friends)?<br>Placing a bet at a betting shop (e.g. on football or horseracing)?<br>Any other gambling (e.g. online gambling)?	No vs yes	

Table S3. Associations between sexual identity and co-occurrence of poor mental health and health risk behaviours (HRBs) in 10,223 adolescents aged 17 years from the Millennium Cohort Study.

	Model 1		Model 2	
	**RRR**	**95% CI**	**RRR**	**95% CI**
No MH & HRB co-occurrence	1		1	
MH & 1 HRB				
**Sexual identity**				
Heterosexual	1		1	
Bisexual	**3.12**	**2.39,4.07**	**2.44**	**1.85,3.20**
Gay/Lesbian	**3.39**	**2.32,4.96**	**3.16**	**2.14,4.68**
**Sex at birth**				
Male			1	
Female			**3.24**	**2.59,4.07**
**Ethnicity**				
White			1	
Ethnic-minority			0.73	0.52,1.02
**Parental income**				
Quintile 1			1.25	0.93,1.69
Quintile 2			1.21	0.90,1.63
Quintile 3			1.13	0.87,1.46
Quintile 4			1.24	0.94,1.64
Quintile 5 (highest income)			1	
MH & 2 HRBs				
**Sexual identity**				
Heterosexual	1		1	
Bisexual	**3.08**	**2.17,4.37**	**2.38**	**1.66,3.42**
Gay/Lesbian	**2.88**	**1.67,4.98**	**2.65**	**1.52,4.64**
**Sex at birth**				
Male			1	
Female			**2.94**	**2.24,3.85**
**Ethnicity**				
White			1	
Ethnic-minority			**0.48**	**0.31,0.74**
**Parental income**				
Quintile 1			1.12	0.77,1.63
Quintile 2			1.34	0.99,1.82
Quintile 3			1.13	0.79,1.61
Quintile 4			0.94	0.66,1.35
Quintile 5 (highest income)			1	
MH & ≥3 HRBs				
**Sexual identity**				
Heterosexual	1		1	
Bisexual	**4.95**	**3.64,6.75**	**4.11**	**2.99,5.66**
Gay/Lesbian	**3.87**	**2.29,6.53**	**3.54**	**2.06,6.08**
**Sex at birth**				
Male			1	
Female			**2.06**	**1.55,2.75**
**Ethnicity**				
White			1	

Ethnic-minority			**0.30**	**0.17,0.53**
**Parental income**				
Quintile 1			**2.36**	**1.49,3.74**
Quintile 2			**1.79**	**1.15,2.77**
Quintile 3			1.37	0.89,2.12
Quintile 4			1.5	0.96,2.34
Quintile 5 (highest income)			1	

MH: Poor mental health (assessed using the SDQ emotional symptoms subscale).
Text in bold indicates statistically significant estimates (95% CI do not contain 1)

Table S4. Associations between co-occurring poor mental health and health risk behaviours (HRBs) and self-harm (or attempted suicide) *excluding* interaction terms, in 10,223 adolescents aged 17 years from the Millennium Cohort Study

	Self-harm				Attempted suicide			
	**Model 1**		**Model 2**		**Model 1**		**Model 2**	
	**OR**	**95% CI**	**OR**	**95% CI**	**OR**	**95% CI**	**OR**	**95% CI**
**No MH & HRB co-occurrence**	1		1		1		1	
MH & 1 HRB	**3.43**	**2.85,4.12**	**3.14**	**2.60,3.79**	**2.09**	**1.60,2.74**	**1.83**	**1.39,2.40**
MH & 2 HRBs	**3.98**	**3.06,5.18**	**3.63**	**2.80,4.72**	**3.13**	**2.29,4.29**	**2.75**	**1.97,3.83**
MH & ≥3 HRBs	**7.62**	**5.60,10.35**	**7.01**	**5.14,9.56**	**8.93**	**6.75,11.82**	**7.8**	**5.82,10.45**
**Sexual Identity**								
Heterosexual	1		1		1		1	
Bisexual	**6.46**	**5.18,8.06**	**6**	**4.81,7.49**	**3.93**	**2.96,5.23**	**3.68**	**2.73,4.96**
Gay/Lesbian	**3.97**	**2.87,5.50**	**3.88**	**2.79,5.38**	**2.36**	**1.49,3.75**	**2.33**	**1.44,3.77**
**Sex at birth**								
Male			1				1	
Female			**1.38**	**1.20,1.58**			**1.75**	**1.41,2.18**
**Ethnicity**								
White			1				1	
Ethnic-minority			**0.73**	**0.60,0.89**			**0.73**	**0.56,0.95**
**Parental Income**								
Quintile 1			1.23	1.00,1.50			**3.25**	**2.39,4.43**
Quintile 2			1.19	0.99,1.42			**2.53**	**1.85,3.46**
Quintile 3			1.03	0.87,1.23			**1.57**	**1.09,2.27**
Quintile 4			1.05	0.87,1.26			**1.44**	**1.02,2.04**
Quintile 5 (highest income)			1				1	

MH: Poor mental health (assessed using the SDQ emotional symptoms subscale). Text in bold indicates statistically significant estimates (95% CI do not contain 1)

Table S5. Associations between co-occurring poor mental health and health risk behaviours (HRBs) and self-harm (or attempted suicide) *including* interaction terms, in 10,223 adolescents aged 17 years from the Millennium Cohort Study

	**Self-harm**				**Attempted suicide**			
	**Model 1**		**Model 2**		**Model 1**		**Model 2**	
	**OR**	**95% CI**	**OR**	**95% CI**	**OR**	**95% CI**	**OR**	**95% CI**
**No MH & HRB co-occurrence**	1		1		1		1	
MH & 1 HRB	**3.46**	**2.79,4.30**	**3.15**	**2.53,3.92**	**2.52**	**1.83,3.47**	**2.1**	**1.52,2.91**
MH & 2 HRBs	**4.63**	**3.44,6.25**	**4.19**	**3.10,5.67**	**3.42**	**2.22,5.27**	**2.92**	**1.86,4.57**
MH & ≥3 HRBs	**8.21**	**5.91,11.41**	**7.5**	**5.37,10.46**	**10.56**	**7.59,14.68**	**9.01**	**6.37,12.76**
**Sexual Identity**								
Heterosexual	1		1		1		1	
Bisexual	**7.22**	**5.64,9.25**	**6.64**	**5.17,8.51**	**4.68**	**3.30,6.62**	**4.19**	**2.91,6.02**
Gay/Lesbian	**3.97**	**2.72,5.80**	**3.83**	**2.61,5.62**	**2.98**	**1.65,5.37**	**2.88**	**1.56,5.29**
**Interactions terms**								
MH & 1 HRB#bisexual	0.76	0.41,1.40	0.81	0.44,1.49	0.58	0.30,1.13	0.67	0.34,1.30
MH & 1 HRB#gay/lesbian	1.55	0.57,4.17	1.6	0.58,4.37	0.58	0.20,1.71	0.63	0.21,1.91
MH & 2 HRBs#bisexual	0.42	0.19,0.90	0.45	0.21,0.98	0.75	0.34,1.65	0.85	0.37,1.93
MH & 2 HRBs#gay/lesbian	0.48	0.15,1.50	0.5	0.16,1.55	0.67	0.15,2.99	0.66	0.13,3.26
MH & ≥3 HRBs#bisexual	0.58	0.25,1.31	0.6	0.26,1.37	0.62	0.29,1.30	0.67	0.31,1.44
MH & ≥3 HRBs#gay/lesbian	0.9	0.24,3.35	0.99	0.27,3.65	0.47	0.14,1.56	0.5	0.15,1.69
**Sex at birth**								
Male			1				1	
Female			**1.37**	**1.20,1.57**			**1.73**	**1.39,2.15**
**Ethnicity**								
White			1				1	
Ethnic-minority			**0.74**	**0.61,0.89**			**0.74**	**0.57,0.96**
**Parental Income**								
Quintile 1			**1.23**	**1.01,1.51**			**3.24**	**2.38,4.40**
Quintile 2			1.18	0.99,1.42			**2.53**	**1.85,3.47**
Quintile 3			1.03	0.87,1.23			**1.58**	**1.10,2.27**
Quintile 4			1.05	0.87,1.26			**1.44**	**1.02,2.04**
Quintile 5 (highest income)			1				1	

MH: Poor mental health (assessed using the SDQ emotional symptoms subscale). Text in bold indicates statistically significant estimates (95% CI do not contain 1)

Table S6. Predicted probabilities for self-harm or attempted suicide based on poor mental health and health risk behaviours (HRBs) co-occurrence and sexual identities in 10,223 adolescents aged 17 years from the Millennium Cohort Study. Estimates are based on multivariable logistic regression models (adjusted for sex, ethnicity and parental income).

	Self-harm			Attempted Suicide		
**Co-occurrence only**	**%**	**95% CI**		**%**	**95% CI**	
MH & 1 HRB	41.7	37.5	45.9	10.1	8	12.3
MH & 2 HRBs	46.7	40.7	52.7	13.8	10	17.6
MH & ≥3 HRBs	61.3	54.3	68.2	30.6	24.9	36.3
**Sexual identity only**						
Heterosexual	20.1	19.1	21.2	6.1	5.4	6.7
Bisexual	58.3	53.3	63.3	18.2	14.3	22.1
Gay/lesbian	46.5	38.6	54.3	13.2	8.1	18.4
Co-occurrence & sexual identity						
No-occurrence & Heterosexual	16	14.9	16.9	4.4	3.8	5.1
No-occurrence & Bisexual	55	49.6	61.1	15.9	11.6	20.2
No-occurrence & Gay/Lesbian	41.9	32.9	50.1	11.7	5.9	17.5
MH & 1 HRB & Heterosexual	37.2	32.4	41.9	8.8	6.5	11.1
MH & 1 HRB & Bisexual	75.7	66.6	84.8	20.1	12.7	28.9
MH & 1 HRB & Gay/Lesbian	77.9	62.1	93.7	14.8	2.8	26.9
MH & 2 HRBs & Heterosexual	44.1	37.1	50.1	11.8	7.4	16.2
MH & 2 HRBs & Bisexual	69.9	57.2	82.6	31.2	18.7	43.6
MH & 2 HRBs & Gay/Lesbian	59.9	35.2	84.6	20.4	1.8	40.6
MH & ≥3 HRBs & Heterosexual	58.3	50.1	66	28.3	21.9	34.8
MH & ≥3 HRBs & Bisexual	84.6	75.1	94.1	51.3	38.1	64.6
MH & ≥3 HRBs & Gay/Lesbian	83.8	67.7	99	36	13.7	58.4

MH: Poor mental health (assessed using the SDQ emotional symptoms subscale). HRB: Health and risk behaviours

Models stratified by sex

S7. Associations between sexual identity and health risk behaviours (HRBs) in 10,223 adolescents aged 17 years from the Millennium Cohort Study. Models stratified by sex (5218 boys and 5005 girls).

	**Boys**				**Girls**			
	**Model 1**		**Model 2**		**Model 1**		**Model 2**	
	**RRR**	**95% CI**	**RRR**	**95% CI**	**RRR**	**95% CI**	**RRR**	**95% CI**
No HRB								
1 HRB								
**Sexual identity**								
Heterosexual	Ref		Ref		Ref		Ref	
Bisexual	1.10	0.66,1.83	1.10	0.66,1.81	**1.31**	**1.00,1.72**	**1.32**	**1.00,1.73**
Gay/Lesbian	1.42	0.77,2.59	1.42	0.77,2.61	**1.67**	**1.04,2.69**	**1.68**	**1.05,2.69**
**Ethnicity**								
White			Ref				Ref	
Ethnic-minority			1.01	0.81,1.25			0.99	0.82,1.21
**Parental income**								
Quintile 1			0.99	0.77,1.28			**1.29**	**1.02,1.62**
Quintile 2			0.97	0.76,1.24			1.21	0.95,1.53
Quintile 3			0.94	0.73,1.22			0.92	0.74,1.14
Quintile 4			0.93	0.73,1.19			0.99	0.81,1.21
Quintile 5			Ref				Ref	
2 HRBs								
**Sexual identity**								
Heterosexual	Ref		Ref		Ref		Ref	
Bisexual	1.31	0.75,2.30	1.24	0.71,2.18	**1.42**	**1.04,1.95**	**1.39**	**1.01,1.90**
Gay/Lesbian	1.37	0.67,2.82	1.36	0.65,2.82	1.51	0.86,2.67	1.47	0.83,2.58
**Ethnicity**								
White			Ref				Ref	
Ethnic-minority			**0.67**	**0.46,0.97**			**0.54**	**0.41,0.70**
**Parental income**								
Quintile 1			**0.66**	**0.48,0.89**			**1.54**	**1.15,2.07**
Quintile 2			0.75	0.55,1.04			**1.35**	**1.04,1.77**
Quintile 3			0.76	0.56,1.03			0.94	0.70,1.26
Quintile 4			**0.70**	**0.50,0.97**			0.92	0.70,1.22
Quintile 5			Ref				Ref	
≥3 HRBs								
**Sexual identity**								
Heterosexual	Ref		Ref		Ref		Ref	
Bisexual	**1.63**	**1.02,2.71**	1.56	0.94,2.59	**2.41**	**1.77,3.29**	**2.32**	**1.70,3.17**
Gay/Lesbian	1.56	0.81,3.00	1.49	0.77,2.90	1.78	0.95,3.33	1.68	0.89,3.16
**Ethnicity**								
White			Ref				Ref	
Ethnic-minority			**0.45**	**0.32,0.64**			**0.28**	**0.19,0.44**
**Parental income**								
Quintile 1			0.81	0.60,1.10			**2.17**	**1.51,3.13**
Quintile 2			0.99	0.72,1.37			**1.77**	**1.25,2.51**
Quintile 3			0.98	0.75,1.28			1.00	0.68,1.47
Quintile 4			1.01	0.75,1.35			1.06	0.73,1.54
Quintile 5			Ref				Ref	

Text in bold indicates statistically significant estimates (95% CI do not contain 1). HRB: Health risk behaviour.

Table S8. Associations between sexual identity and co-occurrence of poor mental health and health risk behaviours (HRB) in 10,223 adolescents aged 17 years from the Millennium Cohort Study. Models stratified by sex (5218 boys and 5005 girls).

	**Boys**				**Girls**			
	**Model 1**		**Model 2**		**Model 1**		**Model 2**	
	**RRR**	**95% CI**	**RRR**	**95% CI**	**RRR**	**95% CI**	**RRR**	**95% CI**
No MH & HRB co-occurrence								
MH & 1 HRB								
**Sexual identity**								
Heterosexual	1		1		1		1	
Bisexual	**3.98**	**2.07,7.64**	**3.82**	**1.98,7.34**	**2.18**	**1.63,2.93**	**2.19**	**1.63,2.95**
Gay/Lesbian	**4.0**	**1.93,8.29**	**3.88**	**1.86,8.08**	**2.85**	**1.82,4.46**	**2.82**	**1.79,4.43**
**Ethnicity**								
White			1				1	
Ethnic-minority			**0.57**	**0.33,0.98**			**0.77**	**0.53,1.12**
**Parental income**								
Quintile 1			0.91	0.50,1.63			**1.44**	**1.02,2.05**
Quintile 2			1.09	0.61,1.96			1.27	0.88,1.82
Quintile 3			0.85	0.50,1.43			1.26	0.93,1.71
Quintile 4			0.99	0.59,1.66			1.35	0.97,1.87
Quintile 5 (highest income)			1				1	
MH & 2 HRBs								
**Sexual identity**								
Heterosexual	1		1		1		1	
Bisexual	**4.61**	**2.08,10.22**	**4.45**	**1.98,9.99**	**2.09**	**1.40,3.10**	**2.02**	**1.36,3.00**
Gay/Lesbian	**5.28**	**2.21,12.62**	**5.23**	**2.18,12.54**	1.93	0.94,3.96	1.85	0.89,3.81
**Ethnicity**								
White			1				1	
Ethnic-minority			0.94	0.45,1.93			0.36	0.21,0.60
**Parental income**								
Quintile 1			0.51	0.23,1.12			1.46	0.95,2.26
Quintile 2			1.20	0.66,2.20			1.40	0.96,2.06
Quintile 3			1.05	0.56,1.96			1.17	0.76,1.81
Quintile 4			0.76	0.38,1.49			1.04	0.69,1.56
Quintile 5 (highest income)			1				1	
MH & ≥3 HRBs								
**Sexual identity**								
Heterosexual	1		1		1		1	
Bisexual	**5.04**	**2.43,10.42**	**4.91**	**2.35,10.26**	**3.91**	**2.77,5.51**	**3.82**	**2.69,5.41**
Gay/Lesbian	**5.95**	**2.63,13.49**	**5.66**	**2.48,12.96**	**2.69**	**1.30,5.57**	**2.55**	**1.22,5.34**
**Ethnicity**								
White			1	.			1	
Ethnic-minority			**0.39**	**0.15,0.98**			**0.27**	**0.13,0.54**
**Parental income**								
Quintile 1			1.19	0.54,2.59			**3.28**	**1.85,5.82**
Quintile 2			1.17	0.57,2.39			**2.22**	**1.28,3.87**
Quintile 3			1.02	0.48,2.18			1.62	0.93,2.80
Quintile 4			1.35	0.66,2.79			1.6	0.91,2.79
Quintile 5 (highest income)			1				1	

MH: Poor mental health. Text in bold indicates statistically significant estimates (95% CI do not contain 1)

Table S9. Associations between poor mental health and co-occurring health risk behaviours (HRB) and self-harm or attempted suicide including interaction terms, in 10,223 adolescents aged 17 years from the Millennium Cohort Study. Models stratified by sex (5218 boys and 5005 girls).

	**Self-harm**				**Attempted suicide**			
	**Boys**		**Girls**		**Boys**		**Girls**	
	**OR**	**95% CI**	**OR**	**95% CI**	**OR**	**95% CI**	**OR**	**95% CI**
**No co-occurrence**	1		1		1		1	
MH & 1 HRB	**3.54**	**2.39,5.24**	**3.1**	**2.40,4.00**	1.77	0.78,4.03	2.08	**1.45,2.98**
MH & 2 HRBs	**3.08**	**1.79,5.29**	**4.69**	**3.28,6.71**	**3.96**	**1.35,11.58**	**2.58**	**1.59,4.21**
MH & ≥3 HRBs	**8.49**	**4.88,14.77**	**7.03**	**4.68,10.56**	**12.73**	**6.93,23.37**	**7.56**	**4.88,11.72**
**Sexual Identity**								
Heterosexual	1		1		1		1	
Bisexual	**4.96**	**3.29,7.48**	**7.59**	**5.62,10.25**	**5.82**	**3.00,11.29**	**3.66**	**2.45,5.49**
Gay/Lesbian	**2.94**	**1.73,4.99**	**4.8**	**2.79,8.25**	**3.81**	**1.66,8.76**	**2.37**	**1.08,5.22**
Interaction terms								
No co-occurrence	1		1		1		1	
MH & 1 HRB#bisexual	1.15	0.32,4.15	0.71	0.36,1.41	0.69	0.15,3.27	0.72	0.34,1.50
MH & 1 HRB#gay/lesbian	2.51	0.50,12.58	1.19	0.34,4.18	1.84	0.28,12.14	0.42	0.10,1.84
MH & 2 HRBs#bisexual	0.57	0.12,2.64	0.4	0.17,0.96	0.48	0.05,4.97	1.02	0.42,2.48
MH & 2 HRBs#gay/lesbian	0.5	0.09,2.84	0.6	0.13,2.71	0.17	0.00,7.67	1.01	0.18,5.80
MH & ≥3 HRBs#bisexual	6.73	0.58,78.66	0.44	0.18,1.08	0.91	0.18,4.60	0.71	0.31,1.66
MH & ≥3 HRBs#gay/lesbian	0.86	0.15,4.89	1.3	0.14,12.34	0.09	0.01,0.94	1.36	0.22,8.29
**Ethnicity**								
White	1		1		1		1	
Ethnic-minority	0.88	0.66,1.16	**0.64**	**0.50,0.83**	**0.54**	**0.31,0.96**	0.82	0.61,1.11
**Parental Income**								
Quintile 1	1.28	0.94,1.73	1.2	0.91,1.59	**3.89**	**2.13,7.07**	**3.05**	**2.13,4.38**
Quintile 2	1.03	0.78,1.37	**1.33**	**1.03,1.73**	**2.52**	**1.43,4.45**	**2.58**	**1.79,3.72**
Quintile 3	0.92	0.70,1.22	1.13	0.89,1.43	**2.04**	**1.13,3.69**	1.43	0.94,2.18
Quintile 4	1.14	0.85,1.52	0.98	0.77,1.26	**1.9**	**1.03,3.49**	1.25	0.85,1.85
Quintile 5 (highest income)	1		1		1		1	

MH: Poor mental health (assessed using the SDQ emotional symptoms subscale). Text in bold indicates statistically significant estimates (95% CI do not contain 1)

Table S10. Predicted probabilities for self-harm or attempted suicide based on poor mental health and health risk behaviours (HRB) co-occurrence and sexual identities in 10,223 adolescents aged 17 years from the Millennium Cohort Study. Estimates are based on multivariable logistic regression models (adjusted for ethnicity and parental income) stratified by sex

Co-occurrence & sexual identity	Self-harm						Attempted Suicide					
	Boys			Girls			Boys			Girls		
**Co-occurrence status**	**%**	**95% CI**		**%**	**95% CI**		**%**	**95% CI**		**%**	**95% CI**	
No co-occurrence	15.9	*14.5*	*17.3*	24.4	*22.6*	*26.2*	3.7	*3*	*4.4*	7.8	*6.7*	*8.9*
MH & 1 HRB	39.6	*31.6*	*47.6*	45.9	*41.1*	*50.7*	6.3	*2.6*	*9.9*	13.2	*10.4*	*16*
MH & 2 HRBs	34.9	*23.4*	*46.4*	53.9	*47.1*	*60.6*	11.6	*2.9*	*20.3*	17.2	*12.7*	*21.7*
MH & ≥3 HRBs	60.3	*48.2*	*72.3*	63.8	*55.8*	*71.7*	28.4	*18.1*	*38.6*	35	*27.6*	*42.3*
**Sexual Identity**												
Heterosexual	16.3	*14.9*	*17.7*	23.8	*22.2*	*25.4*	3.8	*3.1*	*4.5*	8.2	*7.2*	*9.3*
Bisexual	47.4	*38.2*	*56.6*	65	*59.6*	*70.5*	16.4	*9.2*	*23.7*	21.8	*17.3*	*26.3*
Gay/Lesbian	35.5	*24.8*	*46.1*	56.5	*45.7*	*67.3*	11.1	*4.4*	*17.9*	15.8	*8.9*	*22.7*
Co-occurrence & sexual identity												
No-occurrence & Heterosexual	14.1	*12.8*	*15.4*	17.6	*16*	*19.3*	3	*2.3*	*3.6*	5.9	*4.9*	*6.9*
No-occurrence & Bisexual	44.8	*34.9*	*54.8*	61.6	*55.1*	*68.2*	15	*7.2*	*22.7*	18.4	*13.2*	*23.7*
No-occurrence & Gay/Lesbian	32.6	*21.3*	*43.9*	50.5	*37.4*	*63.5*	10.5	*3.3*	*17.7*	13	*4.7*	*21.2*
MH & 1 HRB & Heterosexual	36.7	*28.1*	*45.4*	39.8	*34.2*	*45.4*	5.2	*1.4*	*9*	11.5	*8.5*	*14.5*
MH & 1 HRB & Bisexual	76.5	*56.4*	*96.6*	77.8	*68.4*	*87.3*	17.7	*9*	*34.5*	25	*15.1*	*34.9*
MH & 1 HRB & Gay/Lesbian	80.2	*56.9*	*1.04*	78.5	*59.4*	*97.6*	27	*-1.4*	*55.3*	11.9	*-3*	*24*
MH & 2 HRBs & Heterosexual	33.6	*21.3*	*45.8*	49.9	*41.7*	*58.1*	19	*1.3*	*20.6*	13.8	*8.6*	*19.1*
MH & 2 HRBs & Bisexual	58.5	*25.3*	*91.7*	75	*62.2*	*87.7*	26.2	*-6.3*	*58.7*	36.6	*22.4*	*50.7*
MH & 2 HRBs & Gay/Lesbian	42.6	*4.2*	*80.9*	73.8	*47.5*	*1.002*	12	*20.1*	*44.1*	27.6	*-3*	*55.5*
MH & ≥3 HRBs & Heterosexual	58.1	*45.2*	*71.1*	59.8	*50.5*	*69.2*	27.2	*16.3*	*38.1*	31.2	*22.8*	*39.7*
MH & ≥3 HRBs & Bisexual	97.2	*88.5*	*1.059*	83	*72.6*	*93.5*	64.7	*36.1*	*93.2*	53.3	*39.8*	*66.9*
MH & ≥3 HRBs & Gay/Lesbian	77.5	*50.3*	*1.046*	89.8	*71.6*	*1.08*	12.9	*8.8*	*34.5*	58	*23*	*93.1*

MH: Poor mental health (assessed using the SDQ emotional symptoms subscale).
